# Role of Astrocytes in Post-traumatic Epilepsy

**DOI:** 10.3389/fneur.2019.01149

**Published:** 2019-11-13

**Authors:** Songbai Xu, Qihan Sun, Jie Fan, Yuanyuan Jiang, Wei Yang, Yifeng Cui, Zhenxiang Yu, Huiyi Jiang, Bingjin Li

**Affiliations:** ^1^Department of Neurosurgery, the First Hospital of Jilin University, Changchun, China; ^2^School of Pharmaceutical Sciences, Jilin University, Changchun, China; ^3^Jilin Provincial Key Laboratory on Molecular and Chemical Genetic, The Second Hospital of Jilin University, Changchun, China; ^4^Department of Pediatrics, Yanbian Maternal and Child Health Hospital, Yanji, China

**Keywords:** traumatic brain injury, epilepsy, astrocytes, hyperexcitability, neuron

## Abstract

Traumatic brain injury, a common cause of acquired epilepsy, is typical to find necrotic cell death within the injury core. The dynamic changes in astrocytes surrounding the injury core contribute to epileptic seizures associated with intense neuronal firing. However, little is known about the molecular mechanisms that activate astrocytes during traumatic brain injury or the effect of functional changes of astrocytes on seizures. In this comprehensive review, we present our cumulated understanding of the complex neurological affection in astrocytes after traumatic brain injury. We approached the problem through describing the changes of cell morphology, neurotransmitters, biochemistry, and cytokines in astrocytes during post-traumatic epilepsy. In addition, we also discussed the relationship between dynamic changes in astrocytes and seizures and the current pharmacologic agents used for treatment. Hopefully, this review will provide a more detailed knowledge from which better therapeutic strategies can be developed to treat post-traumatic epilepsy.

## Introduction

Astrocytes, star-shaped glial cells, constitute ~30% of cells in the central nervous system (CNS) (1). They are tightly integrated into neural networks and provide metabolic and physical support for neurons (2). Historically, they have been proposed to participate in blood–brain barrier formation and involved in the maintenance of the extracellular ionic and chemical homeostasis (3–5). Work over the past decade has shown that astrocytes play an important role in the regulation of neuronal development, and plasticity and neuronal hyperexcitability. Recent findings suggest that astrocytes might provide positive feedback to nerve terminals and regulate the release of neurotransmitters (6). The extensions enwrap of astrocytes with presynaptic terminals and post-synaptic spines generate a complex, interconnected hub, the so-called tripartite synapse (7). This may be the dominant form of astrocyte–neuron interactions. Changes in neurons can cause changes in protein and gene expression patterns and morphology of astrocyte (8–10). It is a phenomenon named reactive astrogliosis and characterized by hypertrophy of primary processes, a dramatic increase in the expression of glial fibrillary acidic protein (GFAP), a decrease in expression of glutamine synthetase (11–13). Numerous studies have provided compelling evidence of profound alterations in the morphology and function of astrocytes in epilepsy (14–16). That is, epilepsy can induce reactive astrogliosis. In this context, astrocytes have been implicated in the etiology of epileptic seizures. However, the specific role of astrocytes in epileptic seizures has yet to be fully elucidated.

Traumatic brain injury, a leading cause of acquired epilepsy, is typical to find necrotic cell death within the injury core and reactive astrogliosis surrounding the injury. Approximately 14–20% of patients with moderate to severe traumatic brain injury will suffer from post-traumatic epilepsy (17–19). The risk of post-traumatic epilepsy goes hand in hand with the severity of the trauma. Different from other types of epilepsy, post-traumatic epilepsy with a more complicated etiology is induced by many pathobiological mechanisms in parallel. In order to clarify the extent and exact mechanisms of the astrocyte involved, various kinds of post-traumatic epilepsy-induced traumatic brain injury models have been tested, including controlled cortical impact and the fluid percussion injury models (20), impact-acceleration models (21), weight-drop models, ferric chloride models, and so on (22). Some researchers demonstrated that the loss of astrocyte homeostatic functions possibly contributes to post-traumatic epilepsy (21). But some others reasoned that astrocytes contributing to the intensity of electrical activity is a result of the seizure discharge, rather than underlying the seizure (23, 24). In this article, we summarize the evidence supporting the upstream molecular mechanism inducing astrocyte dysfunction of traumatic brain injury and the downstream physiological consequences of post-traumatic epilepsy in astrocytes. Further, we clarify the interaction between post-traumatic epilepsy and astrocytes.

## Pathophysiology of Traumatic Brain Injury

Falls, motor-vehicle or traffic-related accidents, and blast injury are the causes of traumatic brain injury (25, 26). Outcome from traumatic brain injury is divided into two substantially different stages (27). First, the primary insult involves mechanical tissue deformation occurring at the moment of impact. The most direct result after traumatic brain injury is the tissue damage and impaired regulation of cerebral blood flow. The secondary insult is associated with consecutive pathological processes and the delayed clinical presentation (28). It is characterized by brain edema, metabolic alterations, mitochondrial dysfunction, oxidative stress, excitatory neurotransmitters, and ionic imbalance and activation of inflammatory and immune processes (29). These events lead to functional deficits after traumatic brain injury.

## Pathophysiology of Epilepsy Induced by Traumatic Brain Injury

Many studies have revealed the focal or global cerebral ischemia in brain after traumatic brain injury. This phenomenon leads to the increase in anaerobic glycolysis, decreased glucose uptake, and accumulation of lactic acid. Elevated brain lactic acid levels gradually normalize in patients with milder symptoms, but in fatal traumatic brain injury patients, it still remained heightened than basal levels (30). The post-traumatic brain injury energy crisis will lead to the failure of energy-dependent membrane ion pumps. Traumatic brain injury gives rise to ionic and excitatory neurotransmitters, including acetylcholine, glutamate, aspartate, and a generation of free radicals. The rapid and transient increase in excitatory amino acid signaling has been proposed to become a key factor in the pathophysiology of traumatic brain injury. It has been shown to correlate to injury severity (31–35). This excess in extracellular glutamate results in overstimulation of glutamate receptors with consecutive Ca^2+^, Na^+^, and K^+^ fluxes (36, 37). The release of excitatory neurotransmitters leads to the activation of N-methyl-D-aspartate (NMDA) and a-amino-3-hydroxy-5-methyl-4-isoxazolepropionic acid (AMPA), which further leads to ionic dysregulation (31, 38, 39). Increases of K^+^ amount with injury severity were demonstrated with microdialysis (31). Animal models induced by fluid percussion injury produced a prominent increase in extracellular concentration of K^+^, and it correlates to injury severity. Administration of antagonist of excitatory amino acids attenuated the concentration of K^+^ increase in a dose-dependent manner. In addition, Ca^2+^ accumulation is also commonly observed after traumatic brain injury. The level of Ca^2+^ amount can be elevated in 6 h after the initial injury and be normalized from 4 to 7 days (40, 41). Ca^2+^ activates catabolic enzymes such as lipid peroxidases, proteases, and phospholipases. The sharp rise in Ca^2+^ activity and its decline in the endoplasmic reticulum evoke endoplasmic reticulum-dependent functional disturbances (42). In addition to excitatory neurotransmitters and ionic imbalance observed after traumatic brain injury, the increasing inflammatory events caused by brain insults have predominately been characterized. Notably, changes in several inflammatory cytokines have been observed following traumatic brain injury in human and experimental models (43–45). Intriguingly, interleukin-6 and tumor necrosis factor have been proven to play beneficial roles in promoting regenerative and reparative processes (43, 46). Spontaneous seizures have been found in traumatic brain injury rat and mice models induced by fluid percussion injury and cortical impact injury (47). The restorative changes after traumatic brain injury may be another cause of post-traumatic epilepsy. They are composed of mainly mossy fiber sprouting, rewiring of synaptic circuits, glial cell activation, and ectopic cell proliferation (48, 49). A growing body of evidence has demonstrated the relationship between synaptogenesis process and epileptogenesis (50, 51). The disturbances in the excitatory neurotransmitters and ionic milieu and neuronal synaptic changes lead to epileptogenesis as main factors.

## Reactive Astrocyte Induced by Traumatic Brain Injury

The first modern description of reactive astrocyte was made during the 1970s after the recognition of the GFAP (1), besides the dramatic increase in the length and width of astrocyte soma after traumatic brain injury (52–55). Zhao et al. recently show that a focal damage can cause a global glial reaction (56). The reactive astrocyte is also characterized by the hallmark accumulation of GFAP (57). Changes in the level of GFAP protein and *Gfap* gene are considered to be useful markers for estimating astrocyte reactivity after injury and disease. However, there are some different voices. Some studies indicate that GFAP may not be suitable to be a marker of astrocyte reactivity. It is also expressed by progenitor cells (58). Meanwhile, it is highly heterogeneous in non-mammalian species, rather than a common singular response to injury (59, 60). Serrano-Pozo et al. suggested that in some instances, the higher expression of GFAP is due to higher protein quantities and cortical atrophy, rather than to a proliferation of astrocytes after injury (61). Although GFAP has been a useful biomarker for assessing astrocyte reactivity to date, there remains an unmet need for better markers. Vimentin is another intermediate filament that expressed in astrocytes early in development, but appears to be gradually replaced by GFAP during development (62, 63). Recent transcriptomic studies have revealed some reactive astrocyte genes that might prove to be a better marker for assessing astrocyte reactivity (1). The hypertrophy of reactive astrocytes eventually leads to an increased release of gliotransmitters via volume-sensitive organic anion channels and further enhances network excitability. The reliable biomarkers of astrocyte reactivity may be the marker for the onset of epilepsy after traumatic brain injury.

Besides the most common reaction of astrocytes in morphology, there are some other alternations in reactive astrocyte amounts. Previous research has proved that astrocyte proliferation is limited. After a stab wound injury to the adult brain, the proliferation of astrocyte is about 10% in mouse models (64). Only under the influence of inflammation induced by injection of the bacterial cell-wall endotoxin lipopolysaccharide was there no increase observed in the number of astrocytes in the animal models (9, 65). However, a considerable proliferation of astrocytes is observed after trauma when a protective scar around the injury is produced (66). The glial scar helps to seal off damaged areas and protect healthy brain regions by building a barrier that prevents the infiltration of harmful substances (67, 68). Furthermore, it is thought to prevent recovery from CNS insult by inhibiting neural plasticity, axonal regeneration, and remyelination (69, 70). Thus, astrogliosis and scar may also be involved in regulating neuronal hyperexcitability and seizure development.

Functions of reactive astrocytes are controversial; previous studies show that they can both hinder and support CNS recovery. As we mentioned above, the restorative changes after traumatic brain injury include synaptogenesis. It coincides with hyperexcitability in traumatic brain injury-induced seizures. By contrast, loss of astrocytic processes might stimulate spine sprouting (67). Palisading zone, perpendicular to the injury focus, is characterized by the overlapping processes of adjacent astrocytes. Dendritic spine numbers were reduced in astrocytes of the palisading zone (71) and increased in areas beyond the palisading zone (72). Therefore, they reason that astrocyte processes stabilize and mature the spines they contact. Changes in spine densities appear paradoxical given proliferation of astrocytes in epilepsy. These studies indicate that reactive astrocytes might have layers of different types surround a site of brain insult. In addition, reactive astrocyte phenotypes strongly depended on the type of inducing injury. Zamanian et al. demonstrated that two different types of reactive astrocytes have been induced by neuroinflammation and ischemia, termed “A1” and “A2,” respectively (9). Neuroinflammation-induced A1 reactive astrocytes are more inclined to be destructive to synapses, so the A1 reactive astrocytes might be harmful. A1 astrocytes induced by activated microglia lose most normal astrocytic functions and kill axotomized neurons rapidly after CNS injury (1). Contrary to A1 reactive astrocytes, ischemia-induced A2 reactive astrocytes strongly correlated with promoting survival and synapse repair and up-regulating neurotrophic factors, so they might be protective. Previous studies can also prove this view (73–75). Signal transducers and activators of transcription (STAT)3-mediated proliferate, and neuronal regeneration of reactive astrocytes in animal models is induced by acute trauma (66). Furthermore, A2 reactive astrocytes might be involved with janus kinase (JAK)–STAT3 pathway that helps to control the onset of astrogliogenesis and the later maturation of astrocytes during early brain development and scar-forming (66, 76). Some other studies show that A1 reactive astrocytes might be involved with nuclear factor kappa B cells signaling pathway (1, 76). However, whether this heterogeneity is predetermined remains an unresolved issue.

## Contributions of Astrocytes to Post-Traumatic Epilepsy

**Figure 1:**
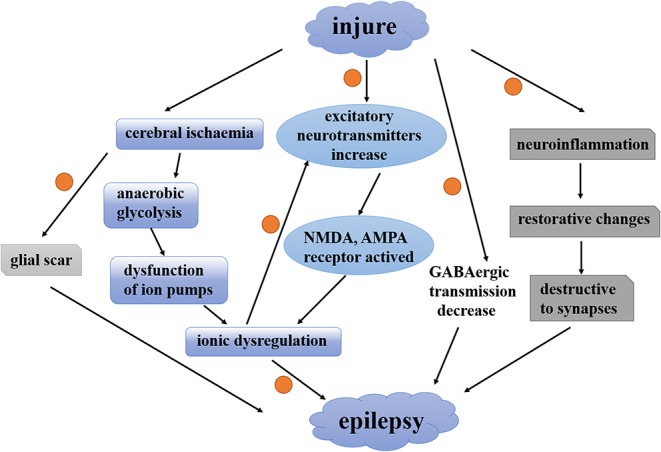
The interaction between astrocytes and epilepsy induced by traumatic brain injury. A contemporary view of how traumatic brain injury induces post-traumatic epilepsy. Orange dots represent sites where astrocytes may be involved in the regulation of post-traumatic epilepsy. NMDA, N-methyl-D-aspartate; AMPA, a-amino-3-hydroxy-5-methyl-4-isoxazolepropionic acid; GABA, γ-aminobutyric acid.

A large body of evidence suggests that astrocytes play an unnegligible role in the epileptic brain. Post-traumatic epilepsy is a long-term negative consequence of traumatic brain injury in which reactive astrocytes can be observed. There are different opinions about the relationship between astrocytes and post-traumatic epilepsy. Since reactive astrocytes can be induced by traumatic brain injury, some researchers reckon that reactive astrocyte is a result of post-traumatic epilepsy while another researcher feels exactly the opposite. To investigate whether astrogliosis is a cause or a consequence of post-traumatic epilepsy, Robel et al. present a mouse model of widespread chronic astrogliosis by conditional deletion of β1-integrin (77). In these mice, astrogliosis occurs in the absence of other pathologies induced by traumatic brain injury. In the meantime, these mice develop spontaneous seizures and brain slices show neuronal hyperexcitability. In addition, gliomas often accompany spontaneous seizures and often escalate to peritumoral epilepsy (78). A glial scar formed by reactive astrocytes is the leading cause of acquired epilepsies. Surgical removal of this glial scar can alleviate the seizures (6). These evidences emphasize the relationship between astrocytes and epileptic seizure. However, the interaction between astrocytes and post-traumatic epilepsy is only partially defined. Traumatic brain injury is characterized by focal or global cerebral ischemia, excitatory neurotransmitters, and ionic imbalance and activation of inflammatory and immune processes (29). Reactive astrocytes induced by these changes can regulate the neuronal plasticity and neuronal hyperexcitability in various ways. Figure 1 shows the interaction between astrocytes and epilepsy induced by traumatic brain injury. This figure mainly shows how traumatic brain injury induces post-traumatic epilepsy. Orange dots represent sites where astrocytes may be involved in the regulation of post-traumatic epilepsy. In this review, we summarize the currently available evidence to show how the molecular and cell biological alterations of reactive astrocytes respond to traumatic brain injury-induced seizures in the following.

### Astrocytes and Neurotransmitters

Several lines of evidence support the notion that astrocytes participate in the extracellular homeostasis of glutamate in the brain. They maintain this function by uptaking the excessive glutamate into astroglial cells efficiently. Traumatic brain injury destroys this function and mediates the elevation of extracellular glutamate concentration. This may be caused by the result of both an increased synaptic release and a deranged glutamate uptake. The high extracellular glutamate levels cause an NMDA receptor-mediated increase in neuronal calcium during seizures, which worsens the seizure phenotype in a continuous loop (79). A study by Tian et al. demonstrates the notion that the AMPA receptor is also involved in it (80). Parpura et al. discovered that the increased calcium levels in neurons induced the release of glutamate from neurons. After this, ATP and D-serine have also been proved to be released from this glia cell (81, 82). Glutamate transporters are required to assure a quick removal of extracellular glutamate. Samuelsson et al. demonstrated that the levels of the astrocytic glutamate transport protein were transient and decrease in an epilepsy model induced by ferrous chloride injection. The level of aspartate transporting protein and β-tubulin III was analyzed at the same time. But they remained unchanged compared to controls (83). These data indicated that the astrocytic glutamate transport protein may be the mechanism of epileptogenesis rather than recurrent seizure. Furthermore, Takahashi et al. suggest that status epilepticus enhanced the capacity of astrocytes to take up extracellular glutamate (84). New research suggests that reactive astrogliosis induces epileptogenesis by impairing the inhibitory action of neuronal γ-aminobutyric acid (GABA) receptors (85). In addition, astrocytosis mediates deficits in inhibitory post-synaptic current-triggered hyperexcitability in hippocampal circuits without altering the intrinsic properties or anatomy of neighboring neurons. This inhibitory post-synaptic current erosion resulted from a failure of the astrocytic glutamate–glutamine cycle. Reactive astrogliosis induced by adenovirus caused the decrease of the glutamine synthetase, which transforms glutamate into glutamine (13). It is essential to provide the rate-limiting precursor for GABA synthesis. Blockade of this enzyme normally induces rapid synaptic GABA depletion. Altogether, these changes lead to the reduction in GABA inhibitory post-synaptic currents. Interestingly, a reduced GABA uptake was found in astrocytes with excessive extracellular GABA levels in a genetic epileptic model (86). Despite the role of GABA in epileptogenesis, controversial aspects still need to be addressed. There is a hypothesis that altered neurotransmitter metabolism in astrocytes can play an important role in seizures. Prof. Steve White identified the two GABA analogs with pharmacological property in preventing seizure activity (87).

### Astrocytes' Function in Ion Homeostasis

As we mentioned above, the level of Ca^2+^ in astrocytes participates in the release of glutamate. Extracellular excessive neurotransmitters acting on astrocyte receptors cause an elevation of cytosolic Ca^2+^. A decrease in Ca^2+^ signals of astrocytes can be observed after injection of anticonvulsants. These evidences indicate that astrocytes participate in the development of epilepsy by regulating the cytosolic Ca^2+^. As we know, the homeostasis of spatial potassium buffering is crucial for the control of neuronal excitability. Kir4.1, an inwardly rectifying potassium channel, is expressed in astrocyte end feet surrounding the CNS and effectively aids in spatial potassium buffering (88, 89). Kir4.1 is down-regulated in epilepsy (90, 91). The loss of Kir4.1 channel function shifts the biophysical properties of reactive astrocytes and increases the resting potential (13, 92, 93). Deficient K^+^ spatial buffering and severe epilepsy were observed in the glial-conditional Kir4.1 knockout animal model (94, 95). In addition, traumatic brain injury can cause the loss of Kir4.1 and Kir2.3 channels in astrocytes (96) and induce post-traumatic epilepsy (97). Takahashi et al. have demonstrated that astrocyte potassium buffering capability remains intact soon after status epilepticus and prior to the development of epilepsy (84). This study indicates that these changes may be the consequence of the epileptic condition rather than a cause. Further investigation of the relationship between astrocyte potassium buffering and network excitability is needed.

### Astrocytes' Function in Water Homeostasis

Water homeostasis is another key mechanism of epileptogenesis in which astrocytes play a pivotal role. Cerebral edema induced by water accumulation in astrocyte is the dominant feature in traumatic brain injury. Marked changes in expression of the astrocyte membrane channels aquaporin-4 (AQP4) have been involved in human and animal studies of epilepsy and traumatic brain injury. A series of studies showed spatial and time overlap of Kir4.1 and AQP4 in astroglial end feet (98, 99). In addition, compared with Kir4.1, AQP4 displays with strong expression in vascular regions. Some studies suggested that the ability of Kir4.1 in carrying out clearance of extracellular K^+^ was weakened if the number of AQP4 channels decreased (100). AQP4 knockout mice with impaired K^+^ buffering and seizures were reported in another study (100). These evidences support the hypothesis that AQP4 and the Kir4.1 channel cooperate in regulating K^+^ homeostasis in the brain. Besides the regulation of K^+^, Ca^2+^ signaling was also induced by AQP4 through P2Y receptors in astrocytes (101). It is difficult to distinguish whether the decrease of perivascular AQP4 channels is a cause or a consequence for epilepsy. Recently, Alvestad et al. suggested that AQP4 mislocalization precedes the chronic phase of seizures (102). Therefore, astrocytic AQP4 and Kir4.1 channel may be potential targets for a novel treatment for epilepsy.

### Astrocytes, Blood–Brain Barrier, and Gap Junctions

Breakdown of the blood–brain barrier is the initial event in traumatic brain injury. Blood–brain barrier breakdown and seizures are closely related. Transient opening of the blood–brain barrier is sufficient to induce epilepsy (103). Serum factors such as albumin can be taken up by astrocytes, which activates the transforming growth factor-β (TGF-β) pathway leading to epileptogenesis (104). In addition, Weissberg et al. show that albumin induces excitatory synaptogenesis through astrocyte TGF-β/ALK5 signaling (105).

The most extensive gap junction with a hydrophilic pore at its center is observed between astrocyte-coupled cells. This pore allows electrical coupling between cells, chemical and metabolic coupling by transfer ions, second messengers, metabolites, and other small molecules (106). Gap junction is also involved in spatial redistribution of K^+^ and glutamate (107). Naus et al. showed an increase in the expression of connexin (Cx)-43 in epileptic patients (108). Tissue excised from epileptic patients exhibit repeated oscillations, suggesting an increase in excitability of these cells. An enhancement in coupling between astrocytes could contribute to epileptogenesis. Interestingly, conditional knockout of the Cx43 protein in astrocytes induces decreased intracellular coupling among astrocytes and increased velocity in the wave of spreading depression (109). With regard to the previous studies, Cx43 mRNA and protein are generally shown to be increased in human (108), whereas findings from animal models are conflicting. No change in mRNA or protein expression of Cx43 or Cx30 was observed in rats with post-traumatic epilepsy (110). The dynamic nature of the connexin protein may be the reason for discrepancies between studies. In conclusion, Cx43 or Cx30 expression studies yield an inconsistent picture of the role of the astroglial network in the development of epilepsy.

### Astrocytes and Inflammation

Traumatic brain injury is associated with a high risk for developing epilepsy and is known to trigger a rapid inflammatory reaction in the brain. The activation of inflammatory pathways seems to play a crucial role in the etiopathogenesis of post-traumatic epilepsy (111–114). Astrocytes release most of the cytokines. Among inflammatory agents that increase and surround a site of brain insult, interleukin-1beta (IL-1β) and TNF (tumor necrosis factor) α are more likely to participate in the etiopathogenesis of post-traumatic epilepsy. IL-1β is a cytokine implicated in NMDA receptor activation. IL-1β enhances NMDA-induced Ca^2+^ influx in a dose-dependent manner. It was abolished by the IL-1β receptor antagonist. Another work suggests that IL-1β receptor 1 mediates the activation of tyrosine kinases to increase the phosphorylation of the NR2A/B subunit and ultimately enhance NMDA-mediated Ca^2+^ influx (115, 116). Apart from NMDA receptors, AMPA receptor was reported to mediate spontaneous seizures due to the action of IL-1β on transient receptor potential vanilloid 1 channels (117). Emerging evidence indicates that IL-1β and TNFα reduce glutamate uptake and increase glutamate release in astrocytes (118–120). Recently, Zurolo et al. reported that inflammation down-regulates the mRNA and protein of Kir4.1 through IL-1β (121). This study establishes a connection between inflammation reaction and potassium hypothesis. The high-mobility group box-1 is an additional inflammatory agent produced by reactive astrocytes and mediated by the toll-like receptor 4 (122–124). In addition, a series of recent work show that inflammatory reaction caused the disruption of the blood–brain barrier (104, 125). Then, a more comprehensive pathway of the blood–brain barrier involved in inducing astrocyte reactivity will further lead to the development of epilepsy. These studies provide strong support to the notion that astrocytes are involved in the inflammatory reaction that favors epileptogenesis.

## Drugs for Preventing Post-Traumatic Epilepsy

Post-traumatic epilepsy is one such common consequence of traumatic brain injury (126–128). It is characterized by a very prolonged latency before epilepsy seizures, which can range from weeks to years (126, 127, 129). The latent period may offer an opportunity to stop or modify the epileptogenic process (130). However, the mechanism at the basis of post-traumatic epilepsy is not completely understood. So, few specific therapies have been developed to prevent post-traumatic epilepsy. Several classic antiepileptic drugs (AEDs) are routinely used in clinical practice, which can only control post-traumatic seizures but cannot prevent the epilepsy seizures. Tetradoxin has been used in the controlled cortical impact model successfully to prevent epileptogenesis (131). Phenytoin and levetiracetam are commonly used as prophylaxis for post-traumatic epilepsy (132–134). Phenytoin is recommended by the American Academy of Neurology (AAN) and Brain Trauma Foundation guidelines for early post-traumatic epilepsy prophylaxis during the first week after a traumatic brain injury, but little to no benefits has been shown in late post-traumatic epilepsy (135). Levetiracetam may be a reasonable alternative to phenytoin for early post-traumatic epilepsy prophylaxis. A recent study showed that phenytoin and carbamazepine, phenobarbital, and the combination of phenobarbital and phenytoin may reduce the incidence of provoked seizures (136). Recent studies demonstrated that mammalian target of rapamycin (mTOR) inhibitors (rapamycin) might have antiepileptogenic effects in the controlled cortical impact model of traumatic brain injury. In addition, rapamycin prevented the development of post-traumatic epilepsy (137). Dual inhibitors of phosphoinositide 3-kinase (PI3K) and mTOR (NVP-BEZ235) could be more effective than rapamycin alone to prevent epilepsy development. Wang et al. have reported the interaction between mTOR pathway in astrocytes and epilepsy. They also found that mTOR deletion from reactive astrocytes prevented increases in seizure frequency (138). Nowadays, astrocytes have been taken into consideration as the potential target for treatment of post-traumatic epilepsy. As we mentioned above, neuroinflammation-induced A1 reactive astrocytes might be harmful. A2 reactive astrocytes might be involved with JAK-STAT3 pathway and scar-forming. So, neutralizing antibodies to TNF and IL-1 can prevent the formation of A1, and drugs targeting TNF and IL-1 may be new targets for treatment of a variety of acute CNS injuries (1). Similarly, drugs that inhibit nuclear factor kappa-B activation in astrocytes or promote STAT3 activation might hold therapeutic potential (1). Taken together, these studies indicate that the signaling pathway alterations in astrocytes should be taken into account as a potential therapeutic target for post-traumatic epilepsy treatment.

## Conclusion

**Figure 2:**
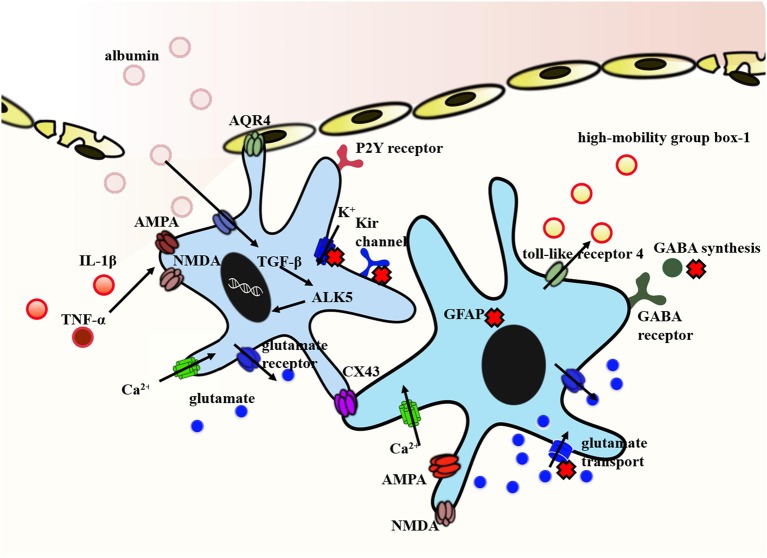
Representative molecular mechanism of post-traumatic epilepsy involved in astrocytes. The exposure of brain tissue to extravasated serum albumin induces activation of TGF-β/ALK5 signaling in astrocytes. IL-1β and TNFα implicated in NMDA and AMPA receptor activation inducing Ca^2+^ influx in a dose-dependent manner. In addition, IL-1β and TNFα down-regulate the expression of Kir channels and weaken its clearance of extracellular K^+^. Decreased expression of Kir channels in concert with dislocation of AQP4 channels in astrocytes contribute to impaired K^+^ buffering. In this context, reactive astrocytes dramatically transform in their morphology and function. Reactive astrocyte is characterized by the proliferation of astrocytes and the hallmark accumulation of GFAP. Traumatic brain injury also causes a deranged glutamate uptake and a decreased GABA release. The high-mobility group box-1 is an additional inflammatory agent produced by reactive astrocytes and mediated by the toll-like receptor 4. Finally, the expression of Cx43 protein is different in human and animal. It is generally shown to be increased in human, whereas findings from animal models are conflicting. Arrows show the flow direction. The red forks represent the disordered functions. Some mechanisms are indicated by abbreviations. GFAP, glial fibrillary acidic protein; NMDA, N-methyl-D-aspartate; AMPA, a-amino-3-hydroxy-5-methyl-4-isoxazolepropionic acid; GABA, γ-aminobutyric acid; AQP4, aquaporin-4; TGF-β, transforming growth factor-β; Cx43, connexin-43; IL-1β, interleukin-1beta.

It is clear that astrocytes are key players in the epileptogenesis induced by traumatic brain injury. A number of astrocyte-specific functions linked to post-traumatic epilepsy have been affected, such as cytokine regulation, water regulation, neurotransmitters and ion homeostasis, and blood–brain barrier maintenance (Figure 2). Part of these changes comes from the disturbance by traumatic brain injury while others are due to the loss of their homeostatic functions. For more than a decade, data have accumulated showing that reactive astrocytes act as “double agents” after traumatic brain injury. On the one hand, they form glial scars that help to seal off injured areas of the brain. On the other hand, these scars may become a seizure focus. In addition, differences in cause of primary brain damage may lead to different effects of reactive astrocytes on spine densities. These studies indicate the complexity of the contribution of reactive astrocytes in post-traumatic epilepsy. Others might speculate that reactive astrocyte is an attempt of the injured brain to repair itself, accompanied by a serious side effect. Anticonvulsant prophylaxis is ineffective at preventing subsequent development of epilepsy nowadays. Thus, there remains an unmet need to explore new antiepileptogenic therapies.

## Author Contributions

SX, QS, JF, YJ, WY, and YC participated in the discussion of the paper and approved the final version of the manuscript for submission. ZY, HJ, and BL provided the critical revisions.

### Conflict of Interest

The authors declare that the research was conducted in the absence of any commercial or financial relationships that could be construed as a potential conflict of interest.

## Abbreviations

CNS: central nervous system
GFAP: glial fibrillary acidic protein
NMDA: N-methyl-D-aspartate
AMPA: a-amino-3-hydroxy-5-methyl-4-isoxazolepropionic acid
STAT3: signal transducers and activators of transcription
JAK: janus kinase
GABA: γ-aminobutyric acid
AQP4: aquaporin-4
TGF-β: transforming growth factor-β
Cx: connexin
IL-1β: interleukin-1beta
TNF: tumor necrosis factor
AEDs: antiepileptic drugs
AAN: American Academy of Neurology
PI3K: phosphoinositide 3-kinase
mTOR: mammalian target of rapamycin.
